# Intraoperative fluorescence redefining neurosurgical precision

**DOI:** 10.1097/JS9.0000000000001847

**Published:** 2024-06-24

**Authors:** Moksada Regmi, Yanni Li, Yingjie Wang, Weihai Liu, Yuwei Dai, Shikun Liu, Ke Ma, Laisan Pan, Jiacheng Gan, Hongyi Liu, Xiuling Zheng, Jun Yang, Jian Wu, Chenlong Yang

**Affiliations:** a State Key Laboratory of Vascular Homeostasis and Remodeling, Department of Neurosurgery, Peking University Third Hospital, Peking University; b Center for Precision Neurosurgery and Oncology of Peking University Health Science Center, Peking University; c Peking University Health Science Center; d Sinotau Pharmaceutical Group; e National Engineering Research Center for Ophthalmology; f Engineering Research Center of Ophthalmic Equipment and Materials, Ministry of Education, Beijing; g Henan Academy of Innovations in Medical Science (AIMS), Zhengzhou, People’s Republic of China

**Keywords:** intraoperative fluorescence, neurosurgery, NP41, visualization

## Abstract

Surgical resection is essential for treating solid tumors, with success largely dependent on the complete excision of neoplastic cells. However, neurosurgical procedures must delicately balance tumor removal with the preservation of surrounding tissue. Achieving clear margins is particularly challenging in cases like glioblastoma due to the limitations of traditional white light visualization. These limitations often result in incomplete resections, leading to frequent recurrences, or excessive resection that harms vital neural structures, causing iatrogenic nerve damage, which can lead to sensory and functional deficits. Current statistics reveal a 90% recurrence rate for malignant gliomas. Similarly, an 8% incidence of iatrogenic nerve trauma contributes to an estimated 25 million cases of peripheral nerve injury globally each year. These figures underscore the urgent need for improved intraoperative techniques for lesion margin and nerve identification and visualization. Recent advances in neurosurgical imaging, such as fluorescence-guided surgery (FGS), have begun to address these challenges. Fluorescent agents used in FGS illuminate target tissues, although not all do so selectively. Despite the promising results of agents such as 5-aminolevulinic acid and indocyanine green, their applications are mainly limited by issues of sensitivity and specificity. Furthermore, these agents do not effectively address the need for precise nerve visualization. Nerve Peptide 41, a novel systemically administered fluorescent nerve-targeted probe, shows promise in filling this gap. This review assesses the major fluorescent imaging modalities in neurosurgery, highlighting each of their benefits, limitations, and potential.

## Introduction

Highlights

- NP41 significantly enhances intraoperative nerve visualization and has shown promising preclinical results, providing a potential strategy to reduce iatrogenic nerve injuries and enhance surgical outcomes.
- Clinical trials have well established the safety and efficacy of FDA-approved fluorescent agents 5-aminolevulinic acid (5-ALA), fluorescein sodium (FNa), and indocyanine green (ICG) in neurosurgery.
- 5-ALA’s role in extending the extent of resection for high-grade gliomas underscores its value in neurosurgery, despite limitations in specificity and depth penetration.
- ICG’s application in cerebrovascular assessment demonstrates its utility in real-time blood flow visualization, aiding in the management of cerebrovascular diseases.
- FNa facilitates the differentiation of malignant brain tumors from surrounding tissues, enhancing the precision of tumor resections.
- A comparative analysis spotlights the distinct advantages and limitations of fluorescent agents, each contributing uniquely to enhanced intraoperative visualization and surgical precision in neurosurgery.

Surgery is the primary treatment for most solid tumors, offering a potential cure if all cancerous cells are removed. Glioblastomas, the most common and challenging tumors in adult neurosurgery, illustrate the limitations of traditional white light methods, which often lead to incomplete resections and risk to adjacent brain tissue^1–3^.

Introduced in 1948, fluorescence imaging began with neurosurgeons using intravenous fluorescein to better visualize intracranial neoplasms^4^. Since then, this technique has proven more effective than white light in several key areas: delineating tumor margins and identifying residual lesions, reducing damage to nerves and vasculature, and evaluating tissue perfusion^5–7^.

This set the stage for subsequent development, and since then, other fluorescent agents like indocyanine green (ICG) and 5-aminolevulinic acid (5-ALA) have been incorporated into various surgical practices^8–13^. ICG, which has been around for over 60 years, found a niche in cancer care in 2005 and, since 2011, has been widely used for visualizing vascular systems during procedures such as arteriovenous malformation (AVM) resections and cerebral aneurysm clippings^14^. 5-ALA, specifically used in high-grade glioma surgeries, significantly improves the extent of resection (EOR) and, consequently, patient survival, marking a landmark achievement in intraoperative fluorescence^15,16^.

Despite their benefits, non-optical imaging methods like intraoperative magnetic resonance imaging (iMRI) present challenges due to their complexity and disruptive nature to surgical workflows. Additional technologies, such as handheld endoscopes and Raman spectroscopy (RS) have been developed; the latter complements FGS by differentiating tissue types through their unique molecular signatures, aiding in precise tumor resection and margin assessment^17,18^. Handheld Raman devices have evolved with enhancements such as miniaturization and Surface-Enhanced RS, becoming more suitable for intraoperative use. These devices now incorporate machine learning to improve the accuracy of tissue classification, thereby enhancing surgical precision. However, despite these advancements, RS faces challenges such as the need for specialized knowledge, high costs, environmental sensitivity, and limited penetration depth, which can restrict its use to superficial tumor assessments^19^. Consequently, FGS, while not without limitations, has continued to grow in use, predominantly as a solitary modality. This growth is evidenced by the substantial increase in related publications, growing from fewer than 50 per year in 1995 to 502 in 2023, and the development of several FGS agents now in clinical trials, such as EC-17, GE3126, and OTL-38^7^.

This review will examine the three established neurosurgical probes and two new ones designed for nerve delineation. Of particular interest is the potential of nerve peptide 41 (NP41) to minimize damage to healthy nerves and ultimately improve clinical outcomes.

## Intraoperative fluorescent agents in practice

### 5-ALA in fluorescence-guided neurosurgery and malignant tumor resection

#### 5-ALA and its mechanism of action

**Figure 1:**
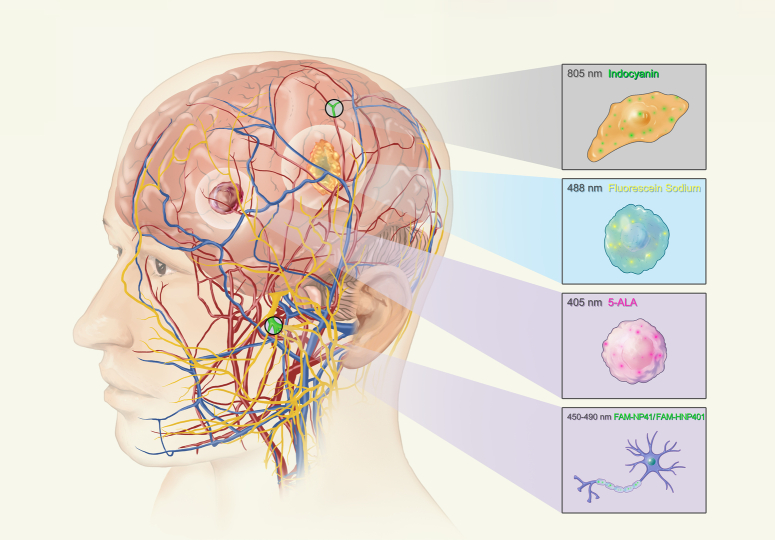
Various fluorescent agents used in neurosurgical visualization. Agents’ names are written in their respective colors of emission. 5-ALA, 5-aminolevulinic acid.

**Figure 2:**
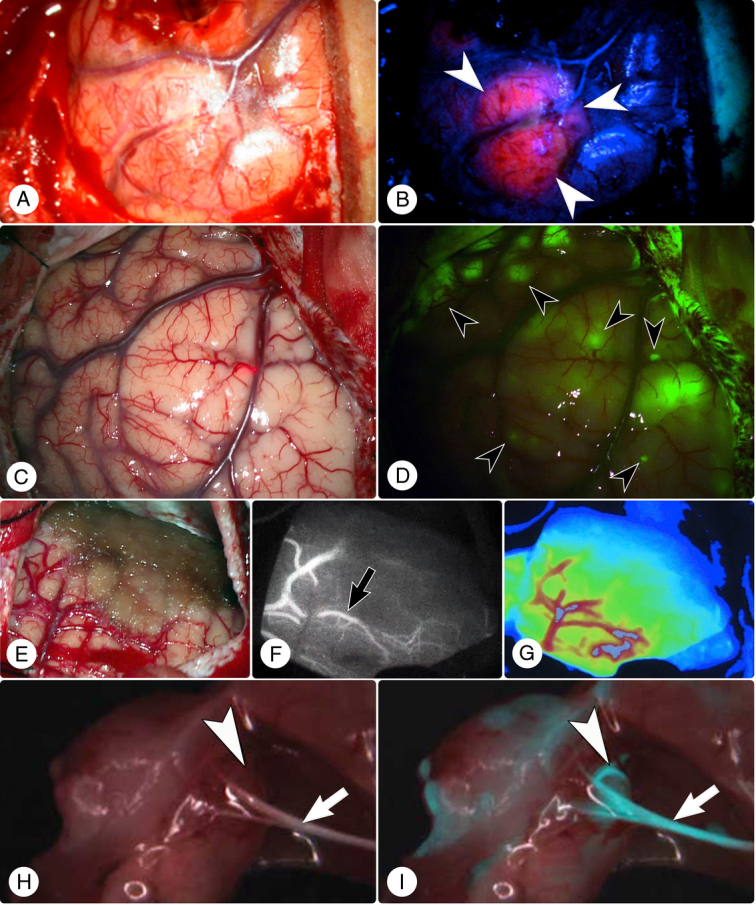
Intraoperative fluorescence imaging using 5-aminolevulinic acid (5-ALA), fluorescein sodium (FNa), indocyanine green (ICG), and nerve peptide 41 (NP41). (A) White light image of the cerebral cortex before 5-ALA administration. (B) Fluorescence imaging with blue light excitation post-5-ALA administration reveals intense red fluorescence in glioma mass (indicated by white arrowheads). (C) The white light image of the cerebral cortex before FNa administration. (D) Enhanced visualization of the tumor region using FNa fluorescence. The fluorescein uptake is preferentially localized to the tumor tissue (black arrowheads), allowing for clear demarcation from the non-tumor tissue. (E) The post-excision white light image of an operative field. (F) ICG angiography reveals patency and flow in cerebral vessels (black arrow) in the resection area. (G) Color-coded Flow 800 visualization of ICG angiography, displaying perfusion levels across the surgical site (blue indicating low perfusion areas and red indicating high perfusion areas). (H, I) The surgical field highlights nerve visibility before (panel H) and after (panel I) NP41 administration. The white arrow indicates a nerve visible in both conditions, while the white arrowhead marks a nerve discernible only post-NP41 application. Panels A and B are courtesy of Ahrens *et al.* (*Cancers*)^20^. Panels H and I are courtesy of Whitney *et al.* (*Nature Biotechnology*)^21^.

5-ALA, a naturally occurring delta amino acid integral to heme synthesis, functions as a prodrug. Strictly speaking, 5-ALA is not classified as a targeted fluorescent agent. However, its mechanism that induces protoporphyrin IX (PpIX) accumulation has found its value in neurosurgery. PpIX is a photosensitive molecule with a proclivity for malignant cell accumulation. Under blue light, PpIX fluoresces (Fig. 1), a property leveraged for FGS, particularly in malignant glioma resections, in which it has proven significant in elevating the EOR (Fig. 2A, B)^22–25^. 5-ALA has been widely used in neurosurgery since its introduction in 2007 in Europe and in 2017 in the United States, and some suggest it to be the only reliable tumor-specific marker^26,27^.

#### Clinical applications and recent advancements

Early in its development, Stummer *et al*.^28^ demonstrated that oral 5-ALA enhances high-grade glioma margin delineation, leading to improved tumor removal and a 6-month progression-free survival rate of 41.0%, compared to 21.1% in white light surgeries. In a large randomized controlled trial by Stenzl *et al*.^29^, involving 814 patients, blue light cystoscopy with the 5-ALA derivative hexaminolevulinate reduced bladder cancer recurrence over 9 months to 47%, compared to 56% with white light only (*P*=0.026). These improvements in surgical outcomes, and lack of significant side effects, made 5-ALA clinically viable for FGS. Since then, 5-ALA has been continually shown to improve tumor resection and almost double complete resection rates of malignant gliomas from 40 to 80% in contrast to conventional white light resection methods, especially in WHO grade 3/4 high-grade glioma cases^30–34^.

Of the recurrences that happen post-glioma excision, over 80% occur within a 2 cm boundary adjacent to the resection cavity^35^. This highlights the challenge of managing infiltrating glioblastoma cells, which can extend beyond the visible tumor lesion. Conventional intraoperative techniques often fail to fully assess invasive tumor growth and the EOR, resulting in residual invasive tumor cells. To address this, the integration of FGS and photodynamic therapy (PDT) offers a strategy. Photosensitizer selection is important in PDT^36–38^. 5-ALA has found a niche in PDT for glioma due to its ability to be selectively taken up by glioma cells and converted into the photosensitive molecule protoporphyrin IX (PpIX)^37^. Once activated by light during PDT, PpIX generates reactive oxygen species that effectively damage and kill cancer cells. This targeted approach helps to visually identify and selectively eliminate tumor tissue, potentially optimizing glioblastoma resection outcomes. Clinical studies have shown that 5-ALA-mediated PDT can significantly enhance the EOR and reduce the rates of tumor recurrence by targeting the residual tumor cells that often evade surgical removal. For example, a study combining 5-ALA PDT with the MEK inhibitor trametinib demonstrated synergistic antitumor effects, significantly reducing cell proliferation and inducing apoptosis in diffuse midline glioma cell lines^39^. Another study highlighted the effectiveness of 5-ALA in sonodynamic therapy, where focused ultrasound and 5-ALA treatment prolonged survival in glioma models by selectively targeting tumor cells^40^.

Recent advancements in 5-ALA neurosurgery have also been bolstered by novel technologies. Scanning fiber endoscopes have been used to enhance the detection of 5-ALA-induced PpIX fluorescence, especially at infiltrative glioma boundaries, outperforming conventional wide-field imaging in sensitivity to low PpIX concentrations^25^. Nanoparticle drug delivery systems and the adoption of laser excitation techniques have further enhanced its precision and achieved complete resection of contrast-enhancing regions^41^. Another notable innovation is the development of a pulsed-light illumination optical system integrated into surgical microscopes^42^. This system allows for the detection of 5-ALA-induced tumor fluorescence without disrupting the surgical process, providing crucial tumor tissue contrast under white light illumination. Integrating 5-ALA FGS with intraoperative imaging has also been shown to improve EOR in high-grade gliomas^30,32,34,43^.

Outside of glioma, studies have demonstrated 5-ALA’s utility in distinguishing metastatic brain tumor tissue from normal brain tissue^44^. While showing less robust fluorescence, its application in meningiomas still contributes to the delineation of tumor margins, aiding surgical strategy^45^. 5-ALA’s use has also been explored in pediatric brain tumors, where it may increase the susceptibility of malignant tumors to PDT^46^. Moreover, the combination of 68-Ga Dotatoc and 5-ALA fluorescence has shown effectiveness in visualizing recurrent skull-base meningiomas, potentially improving resection outcomes, particularly post-microsurgery and Gamma Knife radiosurgery^47^. In spinal tumor surgery, 5-ALA assists in visualizing ependymal tumors, helping to identify residual tumor tissue^48,49^. These expanding applications illustrate 5-ALA’s significant contribution to neurosurgical oncology, although its full potential across various tumor types continues to be explored.

Clinical safety studies confirm the safety of 5-ALA across diverse populations, including children and adolescents^50^. In 2017, the FDA-approved oral Gleolan (5-ALA hydrochloride) as an optical imaging agent for use during surgery in patients with suspected high-grade gliomas, as indicated by WHO grades III or IV on preoperative imaging^51^. This approval marks the first and only time the FDA has specifically approved a drug for glioma visualization. Additionally, its expanding applications, including in conditions like pituitary adenomas, are establishing 5-ALA as a standard in neurosurgical practice^52,53^. Major clinical trials and their results on the safety and effectiveness of 5-ALA, along with other intraoperative fluorescent agents, are listed in Table 1.

**Table 1:** FDA approval and clinical trials of currently used intraoperative fluorescent agents in neurosurgery.

Agent	Approval	Dosage form	FDA-approved derivative	Target	Author	Year	Journal	Country	Clinical trial phase	Enrollment	Comments
5-ALA	2017	Oral	Gleolan	Malignant glioma	Stummer *et al*.^28^	2006	*Lancet Oncol*	GE	III	415	Gleolan increases complete tumor resection (65%) and 6-month progression-free survival (41%) in glioma; major evidence for FDA approval in 2017 (NCT00241670)
					Nabavi *et al*.^54^	2009	*Neurosurgery*	GE	II	40	Gleolan was highly predictive in recurrent glioma surgery, with PPV of 97.2% in pathological areas. Effective across both strong (PPV 91.7%) and weak (PPV 82.4%) fluorescence areas; unaffected by prior treatments
			N.A.	HGG	Vogelbaum *et al*.^55^	2021	*World Neurosurg*	US	N.A.	20	Exoscopic visualization of 5-ALA-induced fluorescence is feasible; it has a high positive predictive value
				Primary solid tumor	–	–	*–*	US	I	130	Unpublished: ClinicalTrials.gov ID NCT04381806
				HGG	Cozzens *et al*.^56^	2017	*Neurosurgery*	US	I/II	33	Safe and effective up to 50 mg/kg for brain tumor fluorescence (NCT01128218)
				Supratentorial brain tumor	–	–	–	GE, NL	II	20	Unpublished: ClinicalTrials.gov ID NCT04738162
				GBM	Michael *et al*.^57^	2019	*J Neurooncol.*	US	I/II	33	Higher doses of 5-ALA FGS were linked to reduced RTV and increased GTR probability; no effect on OS
				Subsurface tumor	Roberts *et al*.^58^	2018	*J Neurosurg*	US	I	540	Detects subsurface tumor fluorescence in most patients, revealing tumors up to 5 mm deep not seen under blue light; less sensitive but effective for depth (NCT02191488)
				Malignant astrocytoma	–	–	–	US	I	6	Unpublished: ClinicalTrials.gov ID NCT01502605
				Malignant glioma	Stummer *et al*.^28^	2017	*Neurosurgery*	DE	I/II	21	20 mg/kg 5-ALA optimal for glioma surgery fluorescence; no effect at 0.2 mg/kg. However, higher doses don’t proportionally increase fluorescence or PPIX levels (NCT02755142)
				Spinal ependymal tumor	Millesi *et al*.^49^	2020	*J Neurosurg*	AUT	N.A.	31	5-ALA fluorescence visualized 81% of spinal ependymal tumors, aiding in detecting unexpected residual tumor in 33% of cases post-GTR
				HGG	Lau *et al*.^59^	2016	*J Neurosurg*	US	II	72	High 5-ALA fluorescence strongly predicts tumor presence (PPV 97.4%) but low NPV (37.7%) for tumor absence. Complication rate 3.4%
				GBM	Cordova *et al*.^60^	2016	*Mol Imaging Biol*	US	II	56	5-ALA FGS in GBM surgery achieved a median EOR of 94.3% and RTV of 0.821 cm^3^, indicating significant tumor burden reduction
				Malignant glioma	–	–	–	CN	III	144	Unpublished: ClinicalTrials.gov ID NCT06160492
				Malignant brain tumor	–	–	–	US	I/II	33	Unpublished: ClinicalTrials.gov ID NCT01128218
				Superficial hemangioma	Zeng *et al*.^61^	2017	*Eur Spine J*	CN	N.A.	181	5-ALA PDL treatment for superficial hemangioma showed a higher complete clearance rate (67.4%) compared to PDL alone (37.0%), with minor adverse effects similar in both groups
				HGG	Jaber *et al*.^62^	2016	*Neurosurgery*	GE	N.A.	166	5-ALA fluorescence predicts higher WHO grade in gliomas, indicated by enhancement and volume
ICG	1959	IV	N.A.	Pelvic nerve	–	–	–	CN	I	86	Unpublished: ClinicalTrials.gov ID NCT05087264
				Glioma	Lee *et al*.^63^	2016	*Neurosurgery*	US	N.A.	15	NIR imaging with second-window ICG accurately localizes gadolinium-enhancing gliomas (sensitivity 98%) but has lower specificity (45%) for margin detection (NCT02710240)
				Cerebral ischemia	–	–	–	US	N.A.	29	Unpublished: ClinicalTrials.gov ID NCT02983786.
				Intracranial meningioma	Lee *et al*.^64^	2018	*J Neurosurg*	US	I	48	Second-window ICG technique shows high sensitivity (96.4%) for intraoperative visualization of meningiomas (NCT02280954)
				Brain tumor	–	–	–	US	I	363	Unpublished: ClinicalTrials.gov ID NCT03262636
				Metastases	Lee *et al*.^65^	2017	*World Neurosurg*	US	I/II	336	SWIG technique allows real-time visualization of brain metastases with high sensitivity (96.4%) but limited specificity
				HGG	Cho *et al*.^66^	2020	*Mol Imaging Biol*	US	N.A.	36	SWIG shows high sensitivity in real-time detection of HGG, with 100% visualization of cortical tumors pre-durotomy
				Intracranial meningioma	Kim *et al*.^67^	2019	*J Neurosurg*	KR	N.A.	42	ICG video angiography effectively monitors blood flow and collateral venous channels in meningioma surgery
				Pituitary tumor	Amano *et al*.^68^	2019	*Acta Neurochir*	JP	N.A.	20	ICG fluorescence endoscopy effectively distinguishes pituitary tumors from normal tissue
				Metastases	Teng *et al*.^69^	2021	*J Neurosurg*	US	N.A.	51	SWIG technique in brain metastases surgery shows strong NIR fluorescence for tumor localization and margin detection, with dose-dependent visibility and improved sensitivity
				Aneurysm	Washington *et al*.^70^	2013	*J Neurosurg*	US	N.A.	155	ICG video angiography shows 75.5% agreement with IA in aneurysm surgery, but 14.3% required clip adjustments after IA due to discordance
				Perfusion measurement	–	–	–	GE	N.A.	30	Unpublished: ClinicalTrials.gov ID NCT01836848
				Spinal nerve	–	–	–	CN	I/II	40	Unpublished: ClinicalTrials.gov ID NCT05808140
FNa	2006	IV	N.A.	Glioma	Chen *et al*.^71^	2012	*Int J Med Sci*	CN	N.A.	22	FNa use increases GTR rates in glioma surgery and prolongs progression-free survival, especially effective in cases with BBB disruption evident on preoperative MRI
				Glioma	Schebesch *et al*.^72^	2018	*Clin Neurol Neurosurg*	GE	N.A.	5	FNa-guided surgery in gadolinium-negative, FET-PET positive gliomas showed precise correlation with metabolic activity, enhancing lesion detection and border identification across various WHO grades. No adverse events were reported
				Nerve identification	–	–	–	US	II	30	Unpublished: ClinicalTrials.gov ID NCT06054178
				Glioma	Xiang *et al*.^73^	2018	*Br J Neurosurg*	CN	N.A.	28	FNa effectively guides resection of HGGs, showing intraoperative fluorescence but not suitable for low-grade gliomas
				Stereotactic brain biopsy	Nevzati *et al*.^74^	2020	*Acta Neurochir*	US	N.A.	19	FNa in stereotactic brain biopsies shows high reliability for identifying tumor pathology in MRI contrast-enhancing lesions
				Spinal cord lesions	Ung *et al*.^75^	2022	*Neurosurg Rev*	US	N.A.	12	FNa demonstrates the potential for enhancing spinal surgery outcomes
				Medulloblastoma	Chen *et al*.^76^	2022	*J Neurooncol*	CN	N.A.	62	FNa usage in medulloblastoma surgery demonstrates safe and effective tumor visualization without side effects
				Pediatric neurosurgical tumor	–	–	–	US	N.A.	2	Unpublished: ClinicalTrials.gov ID NCT03752203
				Spinal intradural tumor	Olguner *et al*.^77^	2021	*Front Oncol*	TR	N.A.	49	FNa guidance under a yellow filter microscope proves beneficial in spinal intradural tumor surgeries, aiding in clear differentiation between tumor and healthy tissue for both intramedullary and extramedullary tumors in 95.9% of cases

5-ALA, 5-aminolevulinic acid; AUT, Austria; BBB, blood–brain barrier; CN, China; EOR, extent of resection; FDA, U.S. Food and Drug Administration; FET-PET, 18F-fluoroethyl tyrosine positron emission tomography; FNa, fluorescein sodium; GE, Germany; GTR, gross total resection; HGG, high-grade glioma; IA, intraoperative angiography; ICG, indocyanine green; JP, Japan; KR, Korea; LGG, low-grade glioma; MB, medulloblastoma; N.A., not applicable/available; NIR, near-infrared; NPV, negative predictive value; OS, overall survival; PDL, pulsed dye laser; PPIX, protoporphyrin IX; PPV, positive predictive value; RTV, residual tumor volume; SWIG, second-window indocyanine green; TR, Turkey; US, United States; WHO, World Health Organization.

#### Limitations and challenges

A primary concern involves the occurrence of false-positive and false-negative results^22,34^. False positives, where non-tumor tissue exhibits 5-ALA-induced fluorescence, can lead to unnecessary resections of healthy structures, a complication intensified by the heterogeneity of gliomas affecting 5-ALA uptake and metabolism^34,78^. Conversely, false negatives, where fluorescence fails in tumor tissue, risk incomplete malignant glioma resections^16^. These inconsistencies in fluorescence detection are further complicated by factors like blood presence, inflammation, and necrosis.

The application scope of 5-ALA is notably limited, primarily effective in guiding resections of contrast-enhancing tumors. It faces challenges with non-enhancing and deep-seated tumors due to reduced fluorescence and limited penetration of blue light in deeper brain tissues^79^. Additionally, its effectiveness is significantly reduced in low-grade gliomas (LGG) because of their lower metabolic activity and proliferative index, which lead to a less pronounced accumulation of PpIX^34,80^. This results in inconsistent visible fluorescence, making the accurate delineation of tumor margins in LGGs challenging^81^. The heterogeneity within LGGs further complicates its application^82^.

While safety considerations are generally favorable, specific risks exist, such as hypotension influenced by cardiovascular conditions and antihypertensive medications, underscoring the need for comprehensive preoperative assessment^83^. Mild transient adverse effects, including phototoxicity, skin rash, and gastrointestinal symptoms, have been documented but are generally considered tolerable^22^.

Economic and logistical factors also complicate 5-ALA’s use in FGS. Variations in cost and accessibility across countries can impede its widespread adoption^22^. Additionally, its inconvenient oral administration hours before use add to these complexities. The risk of skin sensitization within 24 h post-operation, necessitating avoidance of sunlight or strong artificial light, further limits its practicality.

Standardization in the use of 5-ALA in FGS presents an ongoing challenge. Optimal parameters, including dosage, timing, and duration of administration, lack uniform consensus, leading to variances in research and clinical practices. Subjectivity in interpreting 5-ALA-induced fluorescence adds to the variability in assessing the EOR^22^.

In pediatric patients, 5-ALA lacks a well-established safety and efficacy profile. As a result, it is better to refrain from using 5-ALA for FGS in pediatric cases until further research is done.

### Fluorescein sodium: illuminating malignant tumors in neurosurgery

#### Introduction and mechanism of action

FNa, a water-soluble green fluorescent dye, has been utilized in medical applications for over a century^84^. Unlike 5-ALA, which is selectively taken up by glioma cells and metabolized to emit fluorescence, FNa acts through a less specific mechanism. It is administered systemically post-anesthesia and quickly spreads throughout the brain, exploiting disruptions in the blood–brain barrier to non-specifically accumulate in tumor microenvironments (TMEs)^21,85,86^. This results in its concentration not only in tumor cells but also in other cell types within the TME, such as leukocytes, with myeloid cells showing the highest levels of fluorescence.

Under a Yellow 560 nm filter-equipped microscope, FNa provides a clear delineation of malignant brain tumors, allowing surgeons to distinguish between normal and malignant tissue (Fig. 2C, D)^87^. The exact binding targets of FNa remain unclear, suggesting a need for future research to fully understand its binding mechanisms. This will be crucial for refining FNa-guided fluorescence-guided surgery (FGS) techniques and enhancing the precision of tumor delineation and resection.

Despite its non-specificity, FNa’s application as a safe and effective technique for improving CNS tumor visualization and resection is supported by empirical evidence^21^.

#### Recent progress and their clinical implication

FNa’s application in glioma surgery via microscope-integrated fluorescence has proven effective for real-time tumor margin delineation, improving precision and EOR^85,88^. This technique also benefits meningioma surgeries by aiding margin evaluation. Combining FNa with iMRI has further refined glioma surgeries, offering dynamic visualization of tumor boundaries, thus positively impacting EOR and patient outcomes^85,89^. Moreover, FNa coupled with confocal laser endomicroscopy has been used for intraoperative histological imaging, distinguishing tumor from healthy tissue. The FDA-approved ZEISS CONVIVO system, utilizing FNa-based confocal laser imaging, represents a significant advance, providing numerous optical biopsies and real-time histopathological data, surpassing traditional frozen section analysis^90,91^. The EndoScell scanner has followed suit, which can enable real-time in-vivo glioma visualization at the cellular level, with FNa. However, the variability in the fluorescence intensity of the agent has sometimes complicated the interpretation of histological images, affecting the accuracy of tumor margin assessments. With advancing artificial intelligence (AI) technology, integrating image processing algorithms that standardize fluorescence signals could be effective. Additionally, establishing a standardized calibration protocol for FNa, involving precise dosage and administration timing tailored to specific patient and tumor characteristics could further enhance imaging accuracy.

Beyond gliomas, FNa has been used in endocrine adenoma and spinal cord tumor surgeries, improving margin assessment and resection accuracy^21,86^. Furthermore, FNa has been integrated into hybrid operating rooms (ORs), combining conventional surgery with advanced imaging technologies like cone-beam computed tomography, enhancing surgical precision, reducing postoperative imaging needs, and minimizing repeat surgeries^92^. However, the introduction of machines like these in the OR environment raises concerns about increased radiation exposure for both patients and staff, which necessitates rigorous safety protocols and justification of its use based on the risk–benefit ratio in individual cases.

#### Constraints and prospects

FNa presents several limitations and challenges in neurosurgical applications, including its non-specific accumulation in perilesional edema and areas of surgical tissue injury, leading to potential false-positive results^85^. FNa also poses risks of allergic reactions and toxicity concerns. Unlike most fluorescent markers, FNa may cause local or systemic adverse reactions; severe cases, though rare, can be life-threatening^93–95^. Its method of direct injection may also induce mechanical trauma or harm due to the concentrated dye and its carrier. Additionally, FNa’s extended development and blood half-life necessitate early administration before procedures, increasing the risk of immunological reactions^96,97^.

In our clinical observations, FNa persists for a long time in the meninges despite its rapid clearance from the blood. One hypothesis for this prolonged presence is its slow clearance in lymph vessels, which are abundant in the meninges. This suggests that the interstitial fluid may be draining back into the meningeal lymphatic vessels (mLVs), potentially staining the mLVs externally. The question arises whether there are capillary lymphatics involved in this process. The dura mater is a crucial organ for intracranial metabolic waste clearance, and understanding the dynamics of FNa in this context could provide insights into its use as a tracer for these lymphatic pathways. This property could make FNa a useful tracer for these vessels, offering potential new applications in neurosurgical imaging and intervention. Its use for peripheral nerve sheath tumor surgery has been promising, enhancing visualization and EOR^98^. Yet, adoption is limited due to its fluorescence outside the near-infrared (NIR) region.

Beyond these specifications, there are some fundamental problems associated with the use of FNa in neurosurgery that need to be addressed. While FNa is an FDA-approved drug, its approval is specifically for retinal angiography. The application of FNa in neurosurgical procedures, such as for enhancing tumor margins, constitutes ‘off-label’ use and is not legally sanctioned in the strictest terms. Moreover, the recommended dosage and timing for FNa administration are critical for achieving optimal fluorescence; however, our clinical observations have shown no significant difference in surgical outcomes with lower dosages or reduced administration times. Given these concerns, it is necessary to conduct more extensive clinical trials to determine the optimal conditions for FNa use in neurosurgery. With sufficient evidence, these studies could help standardize FNa use and potentially support its FDA certification for neurosurgical applications.

### ICG in neurosurgery: cerebrovascular diseases and beyond

#### Indocyanine and its mechanism of action

ICG, a fluorescent dye utilized since the 1870s, exhibits a strong affinity for plasma proteins, predominantly albumin and α- and β-lipoproteins^99,100^. When administered intravenously, it binds to these proteins and is confined to the vascular compartment due to its high molecular weight. This property allows ICG to be used as a vascular tracer. When illuminated with NIR light, ICG emits fluorescence (Fig. 1), which can be detected by specialized cameras, providing real-time visualization of blood flow and tissue perfusion (Fig. 2E–G)^101,102^.

ICG’s fluorescent properties in the near-infrared spectrum translate to increased tissue penetration and decreased autofluorescence and have given it advantages over other FDA-approved fluorophores, which are mostly in the visible-light spectrum. ICG’s inert nature and binding capacity are pivotal in quantifying blood flow during visceral surgeries, which prompted initial use in colorectal anastomosis^100^. In neurosurgery, its main application in intraoperative fluorescence angiography is for evaluating cerebral aneurysms and cerebral arteriovenous fistulas. It has also been explored to a lesser extent in brain tumor surgery^103–106^.

As early as 1996, Haglund *et al*.^107^ used ICG to enhance the optical imaging of high-grade gliomas. The early 2000s saw a resurgence in the use of FGS for brain tumors, notably following Stummer *et al.*’s 5-ALA trial^28^. This moment renewed interest in ICG’s potential as a visual aid in surgery. Later, in 2012, high-dose ICG imaging, introduced by Madajewski *et al*.^108^, allowed for the detection of residual neoplasms in murine models, setting the stage for the second-window ICG (SWIG) technique developed by our group. By 2016, the first-in-human study revealed significant NIR contrast in gadolinium-enhancing gliomas^63^.

#### Recent advancements

ICG’s utility in neurosurgery has evolved, transcending its initial applications. Intracranial aneurysm management has seen innovations such as microscopic and endoscopic tailored skull-base approaches, intraoperative 3D and ICG video angiography, and advanced aneurysm clip designs, which are already enhancing clinical outcomes in neurosurgery^109–112^.

Notable among new techniques is the SWIG technique, involving preoperative high-dose ICG infusion (5.0 mg/kg) 24 h before surgery. SWIG is utilized in various brain pathologies including high-grade gliomas, meningiomas, brain metastases, pituitary adenomas, craniopharyngiomas, chordomas, and pinealomas^69^.

A significant development is ICG’s integration into microscopes (m-ICG) for real-time blood flow assessment in cerebrovascular surgeries^113^. This innovation has proven effective in aneurysm, AVM, and bypass surgeries, allowing for informed decisions based on dynamic blood flow^114^. Additionally, m-ICG aids in managing pituitary adenomas by evaluating blood flow and demarcating tumor margins, including in endoscopic transsphenoidal surgery for pituitary neuroendocrine tumor tissue. This application has improved resection accuracy and minimized complications^115^. Furthermore, ICG aids in identifying small peritumoral blood vessels in intracranial meningiomas, surpassing standard microvascular Doppler sonography in imaging vessels with lower blood flow or smaller diameter^69^.

In spinal dural arteriovenous fistulas and spinal cord tumors, ICG-assisted blood flow assessment is crucial for identifying fistula sites and tumor margins^116,117^. ICG’s use in cerebral AVMs and aneurysms also aids in evaluating blood flow and identifying essential anatomical features^118^.

Interest in ICG has extended to its role in brain tumor surgery. Developments include shortwave infrared imaging and integration into nanotechnology, leading to ICG nanoparticles for theranostic applications^69^. Park *et al*. have used ICG-loaded liposomes in nanoparticle-mediated photothermal therapy (PTT) for cancer treatment^119^. However, it should be noted that PTT induces an immunosuppressive and pro-inflammatory environment, promoting immune evasion factor recruitment and upregulation. Consequently, we advise against further research in this area until this concern is addressed.

#### Prospects and hurdles

ICG-mediated PDT induces hypoxia, which activates the antitumor activity of co-delivered drugs for a synergistic cell-killing effect. This property makes ICG an excellent agent for exploring combination usage in neurosurgery.

However, one key limitation of ICG video angiography is its restricted field of view, confined to the microscope’s range. This restriction prevents the visualization of anatomical structures outside this scope, which can be crucial during surgical interventions^99,102^. The integration of augmented reality (AR) could help in this context. Additionally, the technique primarily offers qualitative assessments of blood flow without the ability to quantitatively analyze blood supply sufficiency^85,120^. This results in non-standardized interpretations that can vary with the surgeon’s experience. Developing software algorithms capable of quantitatively analyzing fluorescence intensity and dynamics could provide precise measurements of blood flow and tissue perfusion. This could be supported by standardized training protocols using virtual reality to help surgeons learn to interpret ICG angiography images consistently.

Moreover, ICG video angiography sometimes fails to detect neck residuals hidden behind aneurysms, posing a risk of incomplete surgical intervention^102^. A notable challenge also lies in the specialized equipment required for ICG application, which may not be available in all healthcare facilities. Finally, ICG has a short half-life and is rapidly eliminated from the body, often necessitating repeated dosing during procedures^102^. Exploring modified ICG molecules with longer half-lives could reduce the dosing frequency required during procedures, potentially by conjugating ICG with polymers or encapsulating it within nanoparticles to slow its metabolism and excretion.

## GE3126*: advancing visualization in minimally invasive neurosurgery*

### Preliminary evidence

GE3126 (Illuminare-1), developed by Illuminare Biotechnologies is a novel nerve visualization agent designed to augment intraoperative nerve visualization and demarcation. It belongs to a class of diversely substituted styryl-type dyes, synthesized to extend their fluorescent properties toward the red end of the spectrum while retaining drug-like properties and high affinity binding to biological targets^121^. As a single-dose injectable fluorescent compound, GE3126 specifically binds to myelin-basic protein and fluoresces when excited with a light source using a filter centered at 406 nm with a 15 nm bandwidth^121^. Grey literature and reporting suggest it to have improved visualization of nerves, adipose tissue, muscles, and blood vessels as minuscule as ~60 μm, across both open and minimally invasive surgical contexts within 5 min following injection and remained effective for the duration of surgical procedure. Reduced non-specific adipose tissue fluorescence intensity has also been reported, a critical factor in enhancing nerve visibility. However, independent studies have not been performed, and more research with scrutiny is imperative.

#### Clinical applications

GE3126 has not entered clinical usage. In preclinical tests, it has been used in conjunction with a dual-mode (color and fluorescence) laparoscopic imaging instrument to improve the imaging of nerves^121^. This is particularly important in minimal access procedures where small incisions limit visibility. The use of GE3126 has been shown to enhance nerve visibility under fluorescence guidance, especially for small-diameter nerves obscured by fascia, blood vessels, or adipose tissue. In a porcine model, nerve visualization was observed rapidly, within 5–10 min post-intravenous injection, and the nerve fluorescence signal was maintained for up to 80 min^121^.

The safety and efficacy of Illuminare-1 are just being evaluated in a phase I clinical trial in patients undergoing surgery for prostate cancer (NCT04983862). Illuminare-1 has earned the fast-track designation from the FDA.

#### Future

GE3126’s biggest limitation is binding exclusively to the myelin sheath, which is scarce and often absent in many delicate autonomic nerves. However, one interesting way to exploit this drawback would be to use it as a test for myelin degeneration, as the change in intensity of fluorescence can directly suggest a prognosis. Another limitation is the need for blue light illumination, which can be challenging in some surgical settings. Another limitation is the potential for false-positive results, which can occur due to the non-specific binding of the agent to other myelinated tissues. Additionally, the optimal dose and timing of GE3126 administration require further research.

### NP41: bridging the visualization gap in neurosurgery

#### Preliminary evidence

**Figure 3:**
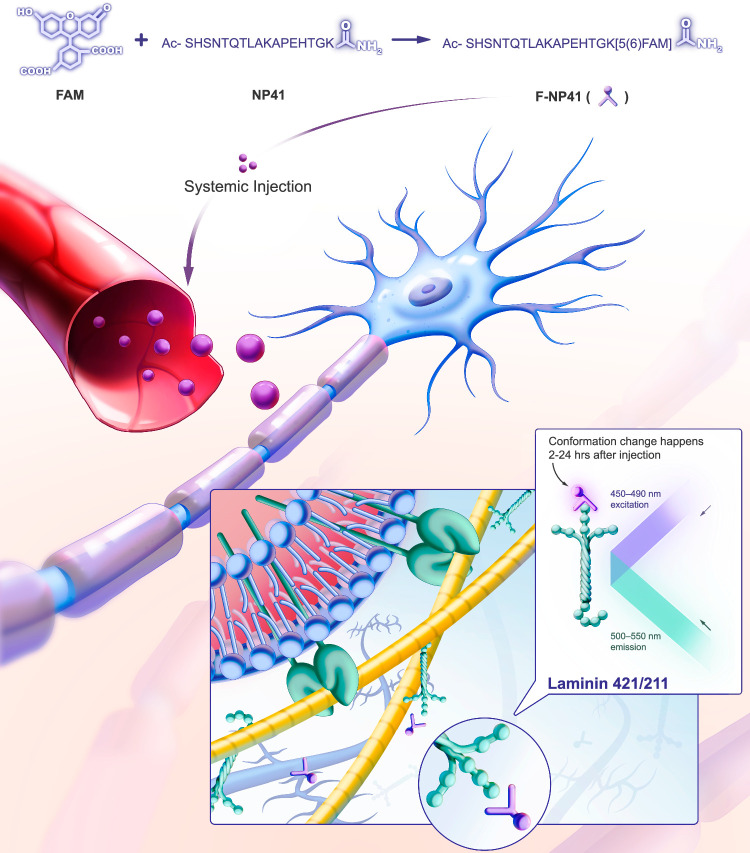
Mechanism of action of nerve peptide 41 (NP41). NP41, tagged with a fluorophore (in this case, FAM), circulates after systemic injection and selectively binds to laminin-421 and laminin-211 within the nervous system’s extracellular matrix. A conformational change occurs 2–24 h post-administration, after which the selective binding appears as cyanish-green (500–550 nm) upon excitation with a 450–490 nm light source.

NP41 is a fluorescently labeled nerve-binding peptide designed to reduce surgical nerve damage and aid nerve repair^122^. After systemic injection, NP41 – tagged with a fluorophore, most commonly FAM or cysteine – circulates and selectively binds to laminin-421 and laminin-211 within the extracellular matrix of the nervous system. A conformational change occurs between 2 and 24 h post-administration. Specifically, when FAM is used as the fluorophore, this change manifests as cyanish-green fluorescence (500–550 nm) under a light source of 450–490 nm, as demonstrated in Figure 3 ^97^.

Histological examinations have identified the nerve connective tissues – epineurium, perineurium, and endoneurium – as primary targets of NP41’s fluorescence^21^. Experiments in murine models have shown that NP41 improves nerve-to-tissue contrast beyond conventional white light imaging, enhancing even the visibility of nerves hidden by overlying tissues, something not achieved by any predecessor (Fig. 2H, I)^97,123^. This facilitates more precise surgical interventions and reduces iatrogenic nerve damage^122,124^.

The increased contrast provided by NP41 allows for the detection of minute, crucial nerve branches, such as the cavernous nerves, which are critical for continence and potency in men and often remain undetected during prostatectomy^97,124,125^.

NP41’s utility extends to identifying severed or degenerated nerves. In situations where nerves lack myelin and axons, NP41 binds to laminins within nerve fibers, a novel approach for highlighting these challenging-to-identify structures^97,123^. This ability also underlines its potential to reveal degenerated nerves months after transection. These remnants, typically invisible to the naked eye, still contain laminins, which NP41 can bind to and highlight. Animal studies have validated NP41’s effectiveness in delineating transected nerves, even months post-injury. Preclinical studies exploring NP41’s potential for nerve visualization, including experiments on various animal models and the specific fluorescent groups attached, are detailed in Table 2. While NP41 has faced criticism for illuminating both degenerated and live nerves, this characteristic can also be seen as advantageous, particularly for facilitating the surgical repair of degenerated nerves^123^.

**Table 2:** Preclinical studies on NP41 for nerve visualization.

Target	Author	Year	Journal	Subject	Fluorescent group attached	Dose	Mode of administration	Equipment	Comments
Facial nerves	Wu *et al*.^96^	2011	*Laryngoscope*	Wild-type albino SKH1 mice	FAM, Cy5	150 nmol/g	IV (tail vein)	Zeiss Lumar fluorescent dissecting microscope /Olympus dissecting microscope	Excellent labeling of both intact and transected facial nerves following NP41 administration. No morbidity or mortality
Facial nerves	Hussain *et al*.^126^	2016	*Laryngoscope*	Female Swiss-Webster mice/Female SKH1 nude albino wild-type mice	FAM	15 nmol/g	IV (tail vein)	Customized Olympus fluorescent dissecting microscope	NP41 fluorescent imaging significantly improved nerve-to-surrounding tissue contrast for both large and smaller buried nerves
Peripheral and autonomic nerves	Hingorani *et al*.^97^	2018	*Theranostics*	Human, Female SKH1 nude albino wild-type mice	FAM	3 mg/kg	IV (tail vein); Topical application	Zeiss Pentero surgical microscope	FAM-HNP-401 showed selective and enhanced binding to human and rodent nerves, enabling improved visualization for potential intraoperative use. Demonstrated safety and efficacy in highlighting peripheral and autonomic nerves without side effects, offering promise for reducing surgical nerve injury
Nerves (non-specific)	Glasgow *et al*.^122^	2016	*PNAS*	Mice (not specified)	FAM	N.A.	IV	Confocal microscopy for ex-vivo tissue analysis; in-vivo fluorescence imaging for nerve visualization	Discovered laminin-421 and laminin-211 as the binding targets of the nerve-highlighting peptide NP41, using a novel receptor capture method and proximity-based labeling
Nerves (non-specific)	Hussain *et al*.^123^	2015	*PLoS One*	Female SKH1 nude albino wild-type mice; female C57/BL6 mice	FAM	15 nmol/g	IV	Customized Olympus fluorescence dissecting microscope for intraoperative imaging, confocal fluorescence microscopy for ex-vivo nerve tissue analysis	FAM-NP41 improved visualization of degenerated facial nerves during surgery in mice, reducing identification time by 39.4% compared to white light. Enhanced post-surgery whisker movement
Peripheral nerves	Whitney *et al*.^21^	2011	*Nat Biotechnol*	Female C57/BL6/SKH1 mice	Fam, Cy5	15–5000 nmol/mouse	IV	Zeiss Lumar fluorescent dissecting microscope, Olympus dissecting microscope, custom-made surgical fluorescence imaging system	FAM-NP41 and Cy5-NP41 selectively labeled all peripheral nerves in mice, providing clear delineation within 2–8 h post-injection without apparent toxicity

Cy5, cyanine5; FAM, carboxyfluorescein; IV, intravenous; N.A., not applicable/available; nmol/g, nanomoles per gram; NP41, nerve-highlighting peptide 41.

NP41 is not typically associated with glioma resection. Resection techniques require clear physical margins, which peptides like NP41 do not provide. However, it holds potential for targeted delivery, like PDT, for nerve vessel bundle-related diseases, such as cerebrovascular malformations. For this, the development of advanced, light-activatable versions of NP41 that can be triggered by externally applied light sources needs to be explored, as the current depth of light penetration remains insufficient for deep-seated vascular lesions. The pharmacokinetics of NP41 must also be optimized to maintain a therapeutic window that maximizes efficacy while minimizing systemic exposure and potential toxicity.

NP41’s diverse applications significantly reduce surgical preparation time by rapidly highlighting all peripheral nerves, mostly within 2 h, minimizing the risk associated with prolonged light exposure^21,123,126^. Further research into NP41’s binding sites could enhance neural recognition accuracy, leading to improvements in binding assays and structure–activity relationships. Clinically, NP41 could improve outcomes for patients undergoing surgical repair of chronic denervation, with its fluorescent labeling speeding up nerve recognition and postoperative recovery^126^. Its ability to recognize minute nerve branches is applicable across a range of surgeries requiring nerve preservation^97^. Additionally, the potential use of NP41-conjugated peptides as carriers for therapeutic agents opens new avenues for exploration^97^.

The recent development of NP41’s successor, HNP-401, presents an even more exciting frontier. Preliminary findings suggest that HNP-401 exhibits notable binding affinity to both motor/sensory and autonomic nerves in humans^97^. Compared to its predecessor, HNP-401 demonstrates a 10-fold increase in signal intensity for binding human nerves, making it an excellent candidate for real-time imaging during surgery^99^. Additionally, HNP-401 showcases a three-fold contrast for nerve-to-muscle on topical sections in human ex-vivo tissue^97^. Its blood clearance profile is similar to NP41, indicating its potential for clinical translation^97^. Furthermore, HNP-401’s specific binding pattern, preferentially binding the perineurium rather than axons, suggests a reduced impact on nerve conductivity^97^. This property makes HNP-401 a promising tool for nerve visualization without affecting nerve function^97^.

To date, due to limited publication, the actual chemical structures of these two probes were not available, making it difficult for researchers to replicate them. We have presented a verified structure of NP41 in Figure 3. However, we are still just testing and verifying the structure of HNP-401.

#### Advantages over traditional nerve-targeting agents

Compared to previous agents, the advantages of NP41 are centered on the following areas:

First, traditional methods of nerve labeling during surgery rely on retrograde or anterograde tracing of individual axonal tracts using fluorescent dyes^122^. These dyes are typically applied to either innervated targets for retrograde labeling or directly to identified nerves for both anterograde and retrograde labeling. However, this local injection approach limits the process to one nerve fiber bundle at a time, posing challenges in preoperative neuroimaging, especially in areas with complex neural networks like the face^21^. NP41 binds to multiple neural tissues concurrently through body fluid transport. This systemic approach overcomes the drawbacks of local injection tracers by labeling all nerves in the body with a single peptide–dye conjugate injection, greatly simplifying preoperative preparations^96^.

Second, traditional neurotracers bind predominantly to axons or myelin sheaths, limiting their application scope. Axonal labeling is inherently restricted as tracers must be transported either retrogradely or anterogradely^21^. This results in only limited labeling along the axonal tracts because retrograde axonal tracers typically accumulate in the neural cell body^21^. Additionally, myelin, either sparse or absent in delicate nerves like autonomic nerves, limits the effectiveness of these dyes in illuminating such critical nerve structures. NP41 targets the extracellular matrix protein layer adhesion protein within the connective tissue surrounding the nerve. This unique binding site allows NP41 to label specialized nerves, including transected, degenerated, and unmyelinated autonomic nerves, addressing the limitations of previous nerve tracers^122^.

Third, NP41 greatly improves the speed of neural visualization compared to previous neural tracers. Retrograde transport is slow, often taking days to label long human nerves like the sciatic nerve and its branches^21^. This delay is incompatible with most surgical procedures. In contrast, NP41 achieves clear contrast of all nerves in the body, including motor and sensory nerves, within just 2–3 h^21,127^.

Fourth, traditional fluorescent dyes used for innervation targets, like direct intramuscular injections to label motor nerves, often result in tracer dye residue at the injection site, impeding accurate observation of adjacent anatomical structures^96^. NP41, however, binds exclusively to characteristic sites around neural tissue, achieving higher neuromuscular contrast and signal-to-noise ratio^21,126^. This specificity enhances the clarity of nerve visualization without the drawbacks associated with previous dyes. The effectiveness, targeting modes, and administration details of fluorescent agents for intraoperative visualization in neurosurgery, including NP41, have been listed in Table 3.

**Table 3:** Comparative analysis of fluorescent agents for intraoperative visualization in neurosurgery.

Agent	Excitation (nm)	Emission (nm)	Targeting mode	Mode of administration	Dosage	Half-life	Time before visualization	Time to fluorescence disappearing in target tissue	GTR	PFS (after surgery)	Sensitivity	Specificity	PPV	NPV	Safety	Cost	Unfavorable features	Favorable characteristics
5-ALA	405	640–710	Metabolic	Oral	20 mg/kg	1–3 h	2–8 h	22 h	69.1%	8 mo	85%	82%	95%	40%	+	+++	Light sensitizer cost	Improves glioma resection rates; enhances progression-free survival
ICG	805	700–850	Passive	IV	0.2–5 mg/kg	3–4 min	Seconds	Several minutes	N.A.	N.A.	~90%	N.A.	N.A.	N.A.	+++	+	Limited tissue penetration; Signal obstruction by blood	Enhances vascular imaging; allows real-time blood flow assessment
SWIG	778	700–850	Passive	IV	2.5–5.0 mg/kg	24 h	>72 h	N.A.	N.A.	N.A.	97%	56%	82%	90%	+	++	Adequate filter, center expertise	Utilizes EPR effect; allows imaging of deep tumors
NP41	450–490 (FAM-NP41), 590–650 (Cy5-NP41)	500–550 (FAM-NP41), 663–738 (Cy5-NP41)	Molecular (laminin-421/211)	IV	15 nmol/g	~10 min (serum), ~50 min to plateau (nerve tissue)	2–3 h (FAM-NP41), 5–6 h (Cy5-NP41)	<24 h	N.A.	N.A.	High	High	N.A.	N.A.	+	++	Limited window of optimal visibility; requires hours for visibility	High contrast for nerve preservation; rapid metabolism; low toxicity; applicability to human tissue observed in *ex-vivo* studies
OTL-38	785	800–835	Molecular (FRα)	IV	N.A.	N.A.	<1 h	N.A.	N.A.	N.A.	High	High	High	N.A.	+	+++	Limited to targeting folate receptor-positive cancers	Targets folate receptor alpha overexpressing tumors
GE3126 (Illuminare-I)	N.A.	N.A.	Molecular (myelin)	IV	0.74 mg/kg	Peak blood fluorescence at 1 h, significant clearance by 4 h	5–10 min	80 min	N.A.	N.A.	High	High	N.A.	N.A.	+	N.A.	N.A.	Improved pharmacokinetics and solubility; rapid visualization post-injection; reduced nonspecific adipose tissue fluorescence
BLZ-100	785	700–850	Molecular	IV	3–30 mg	30 min	3–29 h	48 h	N.A.	N.A.	N.A.	N.A.	N.A.	Headache and nausea are frequent	N.A.	N.A.	N.A.	Targeted tumor imaging
FNa	488	540–690	Passive	IV	2–20 mg/kg	23.5 min	2–4 h	2–4 h	84.4%	7 mo	80.8%	79.1%	98.4%	57.8%	+	+	Atopic reactions, less specific	Visible to the naked eye
IRDye800CW (EGFR)	773	794	Molecular (GRPR)	IV	Up to 24.5 mg/kg	15–20 min	1 h	3–4 d	N.A.	N.A.	88.6% for WHO grade III, 54.7% for WHO grade II	88.2% for both WHO grade III and II samples	95.1% for WHO grade III, 94.6% for WHO grade II	75.0% for WHO grade III, 34.1% for WHO grade II	++	++	Requires preoperative PET imaging for predictive value	Targets EGFR overexpressing tumors

5-ALA, 5-aminolevulinic acid; EGFR, epidermal growth factor receptor; EPR, enhanced permeability and retention; FNa, fluorescein sodium; FRα, folate receptor alpha; GRPR, gastrin-releasing peptide receptor; GTR, gross total resection; ICG, indocyanine green; IV, intravenous; N.A., not available/applicable; nm, nanometer; NP41, nerve-binding peptide 41; NPV, negative predictive value; PFS, progression-free survival; PPV, positive predictive value; SWIG, second-window indocyanine green.

#### Safety profile and toxicity

Animal studies on NP41’s safety show no significant impact on physiological functions or recovery rates post-administration^21,123^. Its low nerve tissue affinity and rapid blood clearance, unlike antibodies, ensure minimal side effects, with almost complete elimination from the body within 24 h^21^

#### Future

Iatrogenic nerve damage contributes significantly to the high recurrence rates of malignant gliomas (90%) and nerve trauma (8%), which are responsible for an estimated 25 million annual cases of peripheral nerve injury worldwide^128,129^. By potentially reducing iatrogenic nerve damage, NP41 could directly improve clinical outcomes in neurosurgery. However, several key issues need addressing before it can be widely used in clinical settings. Firstly, NP41 pharmacokinetics in the human body might differ from murine models, which means adjustments may be needed for optimal use in humans. Secondly, although applying dyes has proven effective with local nerve labeling in animal studies, this approach might be more complicated in human surgeries due to changes in tissue during surgical procedures. Thirdly, improving NP41’s specificity and exploring new ways to deliver it, like using different carrier molecules or controlled-release systems, are crucial. Only clinical trials can answer many of these questions and thus should be judiciously held to comprehensively assess the safety, efficacy, and dosing protocols of NP41. In the beginning, researchers face the challenge of producing statistically significant results, especially when working with small numbers of test subjects.

## Conclusion

FGS enhances the precision of neurosurgical procedures in differentiating and resecting tissues. However, it faces challenges due to the heterogeneity of tumor tissues, limitations of current imaging technologies, and a lack of standardized application guidelines. Advances in FGS rely on the development of targeted, sensitive, and multiplexed fluorescence methods. Additionally, technological innovations such as intraoperative MRI (iMRI), handheld endoscopy, Raman spectroscopy, mixed-reality systems, and dual-modality agents that combine fluorescence with radionuclides are pivotal for advancing personalized and precise neurosurgical oncology. These technological evolutions are set to improve surgical outcomes and patient care, with NP41 marking the initial step in this exciting journey.

## Ethical approval

Not applicable.

## Consent

Not applicable.

## Source of funding

This work was supported by the National Natural Science Foundation of China (82371319 to C.Y.), Beijing Nova Program (20230484356 to C.Y.), Beijing Natural Science Foundation (7222217 to C.Y.), Capital Health Research and Development of Special (2022–4-40918 to C.Y.), AO Spine Research Start-up Grant (AOS-Startup-21–016 to C.Y.), Peking University Clinical Medicine Plus X-Young Scholars Project (PKU2021LCXQ007 to C.Y.), and Peking University Third Hospital Clinical Key Project (BYSYZD2021023 to C.Y.).

## Author contribution

C.Y. and J.W.: conception of the work; M.R., Y.L., Y.W., and Y.D.: design of the work; M.R. and Y.L.: drafted the work; W.L., S.L., K.M., L.P., J.G., H.L., X.Z., and J.Y.: substantively revised the work.

## Conflicts of interest disclosure

The authors declare no conflicts of interest.

## Research registration unique identifying number (UIN)

Not applicable.

## Guarantor

Prof Chenlong Yang, MD, PhD, Department of Neurosurgery, Peking University Third Hospital, 49th Huayuan North Road, Haidian, Beijing 100191, People’s Republic of China, Tel.: +86 13511087060, e-mail: vik.yang@pku.edu.cn; vik.yang@bjmu.edu.cn.

## Data availability statement

This review article does not contain any new data generated by original experimental studies. Instead, it synthesizes and analyzes existing literature and published findings. All sources of information, including previously published studies, reviews, and clinical trials, are duly cited and referenced within the manuscript.

## Provenance and peer review

Not invited; submission was welcomed upon inquiry.
